# p53 mutations in classic and pleomorphic invasive lobular carcinoma of the breast

**DOI:** 10.1007/s13402-012-0071-y

**Published:** 2012-02-22

**Authors:** Cigdem Ercan, Paul J. van Diest, Bram van der Ende, John Hinrichs, Peter Bult, Horst Buerger, Elsken van der Wall, Patrick W. B. Derksen

**Affiliations:** 1 Department of Pathology, H04.312, University Medical Center Utrecht, PO Box 85500, 3508 GA Utrecht, The Netherlands; 2 Division of Internal Medicine and Dermatology, University Medical Center Utrecht, Utrecht, Netherlands; 3 Department of Pathology, Radboud University Nijmegen Medical Centre, Nijmegen, The Netherlands; 4 Institute of Pathology, Paderborn, Germany

**Keywords:** Breast cancer, p53, Mutation, Invasive lobular breast cancer, Classic, Pleomorphic

## Abstract

**Background:**

p53 is a tumor suppressor that is frequently mutated in human cancers. Although alterations in p53 are common in breast cancer, few studies have specifically investigated *TP53* mutations in the breast cancer subtype invasive lobular carcinoma (ILC). Recently reported conditional mouse models have indicated that functional p53 inactivation may play a role in ILC development and progression. Since reports on the detection of *TP53* mutations in the relatively favorable classic and more aggressive pleomorphic variants of ILC (PILC) are rare and ambiguous, we performed a comprehensive analysis to determine the mutation status of *TP53* in these breast cancer subtypes.

**Methods:**

To increase our understanding of p53-mediated pathways and the roles they may play in the etiology of classic ILC and PILC, we investigated *TP53* mutations and p53 accumulation in a cohort of 22 cases of classic and 19 cases of PILC by direct DNA sequencing and immunohistochemistry.

**Results:**

We observed 11 potentially pathogenic *TP53* mutations, of which three were detected in classic ILC (13.6%) and 8 in PILC (42.1%; *p* = 0.04). While p53 protein accumulation was not significantly different between classic and pleomorphic ILC, mutations that affected structure and protein function were significantly associated with p53 protein levels.

**Conclusion:**

*TP53* mutations occur more frequently in PILC than classic ILC.

## Introduction

The tumor suppressor p53 was first described in 1979 as a key cell cycle regulator. Upon cellular stress, the p53 signaling pathway turns on the expression of genes including inhibitors of cell cycle, DNA repair, and apoptosis [1, 2]. Inactivating alterations in the p53 gene are commonly observed in human cancers, resulting in suppression of the regulatory functions of p53 which contributes to transformation of cells. Mutations in p53 are observed in breast cancer, however with a lower frequency (~ 20%) compared to other solid tumors [2]. Although it has been well established that p53 mutations correlate with high grade and triple negativity [3, 4], the p53 mutation spectrum across the various different histological types of breast cancer has not been well defined.

Invasive lobular cancer (ILC) accounts for approximately 15% of breast cancers [5]. Based on their molecular profile, most ILC belong to the luminal–type breast cancers. Within ILC, several subtypes can be discerned: 1) the classic type composed of small regular cells with frequently intracytoplasmic vacuoles, small nuclei and a typical trabecular infiltration pattern with dissociated cells or forming single files, often in targetoid patterns around uninvolved ducts and with low mitotic rate; 2) the better demarcated alveolar type exhibiting small round aggregates of 20 or more cells with typical lobular cytology; 3) the also better demarcated solid variant consisting of more solid sheets of cells with little intervening stroma, more mitoses and often some more atypia; 4) the pleomorphic variant that exhibits the growth pattern of classical lobular carcinoma throughout but with polygonal, eccentric pleomorphic nuclei and more frequent mitoses [6]. The pleomorphic variant of ILC (PILC) accounts for less than 1% of all breast carcinomas and not more than 10% of all ILC [7]. It has a poorer prognosis compared to classic ILC [5, 8]. Several studies have addressed the molecular and histological aspects of PILC as a separate entity. Immunohistochemical analyses demonstrated that PILCs frequently express estrogen (ER) and progesterone (PR) receptors and are mostly E-cadherin negative, Her2 positive and occasionally p53 positive [8–12]. Although expression of gross cystic disease fluid protein (GDCFP) has been used to facilitate differential diagnosis between classic ILC and PILC, reliability is variable [11–14]. Little is known about p53 mutation status in PILC. In view of the higher nuclear grade and poorer prognosis, one would expect a higher frequency of p53 mutation in PILC. Interestingly, conditional knock-out mouse models have indicated that functional inactivation of p53 may play a role in carcinogenesis and progression of mouse PILC [15, 16]. In the present study, we have investigated the p53 mutation status in a series of 41 ILC cases including 22 classic and 19 pleomorphic subtypes to advance current knowledge on these variants of ILC.

## Materials and methods

### Patients

Archival material from 41 breast cancer patients with lobular carcinoma was collected from the Pathology departments of the University Medical Center Utrecht, Utrecht, The Netherlands and Radboud University Nijmegen Medical Centre, Nijmegen, The Netherlands, and the Institute of Pathology, Paderborn, Germany. ILC and PILC were identified on Hematoxylin and Eosin (H&E) stained reference slides from formaldehyde-fixed paraffin embedded breast cancer tissue blocks of 41 cases by an experienced pathologist (PJvD), considering cases with nuclear atypia score 3 as PILC. Use of anonymous or coded left over material for scientific purposes is a part of the standard treatment contract with patients in our hospitals [17].

### DNA extraction

After de-paraffinization of the slides by standard methods, the relevant area from each slide (as identified on corresponding H&E stained sections) was scraped off with a scalpel and suspended in lysis buffer (Tris/HCl buffer pH 8.0 with Tween). Then, proteinase K was added and the samples were incubated for 1 h at 56°C. After that, samples were incubated at 95°C for 10 min to inactivate proteinase K.

### Sequencing and mutation analysis

For the detection of mutations, genomic DNA was amplified with primers flanking exons 4, 5, 6, 7, 8 and 9 of the TP53 gene (Table 1). The PCR conditions were set up as follows; initial denaturation at 94°C for 3 min, 35 cycles at 94°C for 1 min (denaturation), 60°C for 30 s (annealing) and 72°C for 45 s (extension), followed by a final extension at 72°C for 5 min. Then, PCR products were sequenced in both sense and antisense directions using the BigDye Terminator v1.1 sequencing kit on ABI 3130 (Applied Biosystems, Foster City, CA, USA) according to the manufacturer’s instructions. The sequences were analyzed using Mutation Surveyor software (SoftGenetics,LLC., State College, PA, USA).

**Table 1:** Summary of primer sequences

Exon	Sequence
4.1 FW	5′ CTG GTC CTC TGA CTG CTC 3′
4.1 RV	5′ GAC AGA AGA TGA CAG GGG 3′
4.2 FW	5′ AGC TCC CAG AAT GCC AGA G 3′
4.2 RV	5′ TGA AGT CTC ATG GAA GCC 3′
5.1 FW	5′ CCG TGT TCC AGT TGC TTT ATC 3′
5.1 RV	5′ GCT GTG ACT GCT TGT AGA TG 3′
5.2 FWa	5′ TCA ACA AGA TGT TTT GCC AAC TGG 3′
5.2 FWb	5′ ACA AGA TGT TTT GCC AAC TG 3′
5.2 RVint	5′ GAG CAA TCA GTG AGG AAT CAG 3′
6 FW	5′ TCC CCA GGC CTC TGA TTC 3′
6 RV	5′ CTG ACA ACC ACC CTT AAC C 3′
7 FW	5′ CTT GCC ACA GGT CTC CCC AA 3′
7 RV	5′ GCG GCA AGC AGA GGC TGG 3′
8 FW	5′ CCT TAC TGC CTC TTG CTT C 3′
8 RV	5′ TAA CTG CAC CCT TGG TCT C 3′
9 FW	5′ GTT ATG CCT CAG ATT CAC T 3′
9 RV	5′ TGA GTG TTA GAC TGG AAA C 3′

### Immunohistochemistry

Four μm thick sections were cut from the paraffin blocks and transferred to Superfrost + slides (Menzel and Glaeser, Braunschweig, Germany). Citrate buffer was used for antigen retrieval. Immunohistochemistry was then performed with a mouse monoclonal p53 antibody, clone BP53-12-1, 10 mg/mL stock, (MU 195-UC, BioGenex, San Ramon, CA, USA; 1:100) on an automated immunostainer (Bond-MaX, Leica, Bannockburn, IL). Slides were counterstained with hematoxylin, dehydrated, and cover-slipped. Throughout the immunohistochemical analysis, negative controls were obtained by omitting the primary antibody and staining of a colon cancer tissue with a verified p53 mutation was used as a positive control. Scoring of the stained nuclei was performed by an experienced pathologist (PJvD). p53 was regarded as accumulated when ≥5% of nuclei were stained. p53 was regarded as wild-type when approximately 1% of nuclei showed expression.

### Statistical analysis

Chi-Squared test, or, when appropriate, Fischer’s Exact test was applied to compare frequencies between groups with the SPSS 15.0 software package (IBM), regarding p-values <0.05 as significant.

## Results

We identified the pleomorphic ILC variant using H&E staining, based on a classical trabecular ILC growth pattern, but with polygonal eccentric pleomorphic nuclei and more frequent mitoses (cases with nuclear atypia score 3). We have not observed a significant increase in HER2 expression in PILC when comparing with classical ILC in our samples (data not shown).

The rationale behind our investigation of p53 in classic ILC and PILC is gaining a new insight into distinctive gene alterations which give rise to these two different subtypes of ILC.

### p53 mutations

To investigate the incidence of *TP53* mutations in classic ILC and PILC, we performed PCR on exons 4–9 (conserved midregion) of *TP53* for 41 ILC cases (22 classic ILC and 19 PILC). Direct DNA sequencing was subsequently performed on PCR products. Overall, we detected 11 mutations (of which 1 novel and 10 previously reported) and 2 validated polymorphisms in 41 ILC cases (Tables 2 and 3). One out of 11 mutations was located in an intron and 10 mutations were located in coding regions. Using the freely available IARC TP53 database, we have scrutinized the following; the functions of the domains in which the mutated residues are located, the known functions of the wild-type residues, the effect of the mutations, the predicted effect on splicing, functional predictions based on the structure change and previously reported tumor sites (Table 2) [18, 19]. This data summarized in Table 2 allowed us to predict the pathogenicity of the observed mutations. We conclude that all of the 11 mutations found could potentially be pathogenic based on the mentioned criteria above.

**Table 2:** p53 mutation analysis results of Classic and Pleomorphic Lobular Breast Cancer [18] (Version of the database; R15, November 2010)

Case	Location	Genomic description	AA change	Domain function^a^	Residue function^a^	Effect^c^	Predicted effect on splicing^d^	Structure-function^e^	Observed tumor Sites	p53 expression^f^
ILC-1	Exon5	g.12368C>A	p.S127Y	DNA Binding	Buried	Missense	No significant change	Nonfunctional	Mainly Gallbladder, incl. Breast.	100%
ILC-2	Exon6	g.12696A>T	p.R209S	DNA Binding	Exposed	Missense	New Site	Functional	Mainly Liver	1%
ILC-3	Exon6	g.12683A>G	p.Y205C	DNA Binding	Buried	Missense	New Site	Nonfunctional	Mainly in Urinary Tract, incl. Breast	67%
PILC-1	Exon5	g.12741G>A	p.E224E	DNA Binding	Exposed	Silent	Site Broken—New Site	NA	Mainly Bones	1%
PILC-2	Exon8	g.13798C>T	p.R273H	DNA Binding	DNA Binding	Missense	No significant change	Nonfunctional	Mainly Lungs, incl. Breast	20%
PILC-3	Intron7	g.13440C>T	–	NA	NA	Intronic	No significant change	NA	Mainly Nasal Cavity	0%
PILC-4	Exon5	g.12492C>T	p.H168H	DNA Binding	Partially Exposed	Silent	No significant change	NA	Mainly Cervix Uteri, incl. Breast	3%
PILC-5	Exon5	g.12469G>A	p.A161T	DNA Binding	Buried	Missense	No significant change	Nonfunctional	Mainly in Meninges, incl. Breast	30%
PILC-6	Exon5	g.12442C>T	p.P152S	DNA Binding	Partially Exposed	Missense	No significant change	Nonfunctional	Mainly in Renal pelvis, incl. Breast	7%
PILC-7	Exon4	g.11387A>G	p.Q52Q	NA	NA	Silent	New Site	NA	–	3%
PILC-8	Exon5	g.12543C>T	p.S185S	DNA Binding	Exposed	Silent	New Site	NA	Mainly Head&Neck	4%

^a^Domain Function; Function of the domain in which the mutated residue is located

^b^Residue Function; Known function of the wild-type residue (NA = Not Available)

^c^Effect; Effect of the mutation. The terms occurring in this column are: missense (change of one amino-acid) and silent (no change in the protein sequence)

^d^Predicted Effect on Splicing; This is the predicted effect of the mutation on splicing based on two splicing prediction tools NNSPLICE0.9 and HSF V2.3. No significant change: no change in the wild type splice motif. New site: the mutation created a splice motif not present in the wild type sequence. Site broken: the mutation removed a splice motif that was identified in the wild type sequence

^e^Structure-Function; Functional predictions derived from a computer model that takes into account the 3D structure of wild type and mutant proteins and is trained on the transactivation dataset from Kato et al. [19]. Mutations are classified as “functional” or “non-functional”

^f^p53 Expression; The results of immunohistochemistry analysis of p53 protein expression for these cases is represented on this column. (Percentage of cells expressing p53)

**Table 3:** p53 mutation analysis results of Classic and Pleomorphic Lobular Breast Cancer [18] (Version of the database; R15, November 2010)

Number	Location	Genomic description	Aminoacid change	Domain function^b^	Effect^c^	Predicted effect on splicing^d^	Observed in
1	Exon4	g.11339G>A	36P>P	Transactivation	Silent	NA	3 PILC and 3 ILC cases
2	Exon4	g.11446C>G^a^	72P>R	SH3-like/Pro-rich	Missense	New Site	11 PILC and 18 ILC cases

^a^Several studies showed its association with breast cancer in different populations

^b^Domain Function; Function of the domain in which the mutated residue is located

^c^Effect; Effect of the mutation. The terms occurring in this column are: missense (change of one amino-acid) and silent (no change in the protein sequence)

^d^Predicted Effect on Splicing; This is the predicted effect of the mutation on splicing based on two splicing prediction tools NNSPLICE0.9 and HSF V2.3. No significant change: no change in the wild type splice motif. New site: the mutation created a splice motif which is not present in the wt sequence. Site broken: the mutation removed a splice motif that was identified in the wt sequence. (NA = Not available)

Next, we evaluated the distribution of these potentially pathogenic mutations over classic and pleomorphic ILC variants. Eight of the 19 PILC cases (42.1%) exhibited a potentially pathogenic mutation which is significantly more often when compared to the percentage of potentially pathogenic mutations found in classic ILC cases (3 mutations (missense) observed in 22 classic ILC cases (13.6%; *p* ≤ 0.05)) (Table 4). We have also observed two previously reported and validated polymorphisms among our samples. These were distributed similarly over the classic and pleomorphic ILC variants (Table 3).

**Table 4:** Correlation of potentially pathogenic p53 mutations with classic and pleomorphic invasive lobular carcinomas

	Potentially pathogenic p53 mutations		
	+	−	
Classic ILC	3 (13.6%)	19 (86.4%)	Total 22
PILC	8 (42.1%)	11 (57.9%)	Total 19

*p* < 0.05 *

### p53 expression and accumulation

**Fig. 1:**
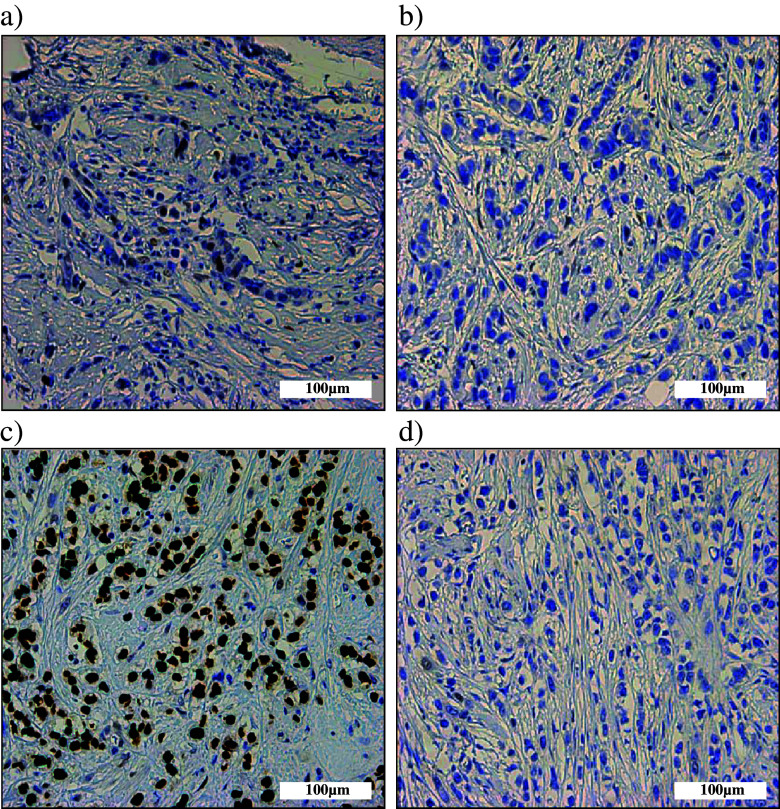
p53 expression in PILC. **a-d**. Representative examples of p53 expression of PILC cases (**a**) wild-type; 1% positive nuclei (**b**) no expression and absence of mutations (**c**) ‘g.12368C>A’ mutation resulting in 100% p53 overexpression (**d**) ‘g.13440C>T’ mutation occurring in an intron site and resulting in absence of p53 expression

p53 immunostaining was performed to investigate the correlation of mutational status and effects on protein expression (Fig. 1). Immunohistochemistry scores and mutation data of each case are summarized in Table 2. We observed a variation in the immunohistochemistry scores of p53 in both pleomorphic and classic cases. In normal breast tissue, p53 staining is observed in a small percentage (approximately 1%) of the cells. Therefore, 5% positivity was chosen as a value to distinguish normal, wild-type p53 expression and mutated p53 overexpression. Overall, 9/41 cases (21.9%) showed p53 accumulation, while 6 cases showed absence of expression. Of these 15 cases, 6 were ILC and 9 PILC (*p* = 0.157). In conclusion, p53 accumulation was not associated exclusively with a specific ILC variant.

### Correlation of p53 accumulation and mutation

Because a large body of literature has shown that p53 accumulation can be caused by mutations in *TP53*, we set out to investigate the correlation between p53 accumulation and mutation. In total, 5 out of 9 cases that showed p53 accumulation based on immunohistochemistry harbored mutations (Table 5). Interestingly, we detected potential pathogenic mutations in 4 of the 32 cases without p53 accumulation (wild-type staining + 0% staining) (*p* = 0.01), while 1 of 6 cases showed a pathogenic mutation in the absence of p53 staining (0% staining). However, the 5 remaining cases with 0% staining had one of the two validated polymorphisms (Number 2 in Table 3). In summary, we observed a significant correlation of p53 accumulation with potentially pathogenic mutations (Table 5).

**Table 5:** Correlation between p53 mutations that give rise to a non-functional structure and p53 accumulation

		Mutations causing a predicted defect in p53 structure	
		−	+
p53 Accumulation	−	32	0
	+	4	5

*p* ≤ 0.001*

## Discussion

Pleomorphic ILC was first described in 1982 as a variant of infiltrating lobular carcinoma [20]. Even though the morphology of PILC with high nuclear atypia is distinctive, its feature of high frequency of multicentrity and bilaterality seem to be similar to the classic ILC [21]. The report of Weidner and Semple [9] is one of several reports demonstrating the aggressive clinical behavior and poorer prognosis of patients with PILC in comparison to patients with ILC [9]. Therefore, despite the similarities between them, the question mark on the genetic pathways through which each variant evolves still remains. The *TP53* tumor suppressor gene has been an interesting target to investigate in invasive breast cancer since it is very frequently altered in other human cancers [22]. Many research groups have investigated the distribution of p53 mutations and its correlation with immunohistochemistry in invasive carcinomas [23–27] but data focusing on different variants of ILC are limited. Therefore our aim was to study the mutational status of p53 in classic and pleomorphic ILC to gain a better understanding of the molecular changes occurring in this gene, which possibly contribute to the development of these subtypes and the potential of it as a tool to differentially diagnose ILC *versus* PILC.

In the present work, we studied 41 ILC cases for p53 mutations and accumulation in relation to the classic and pleomorphic variants. Eleven mutations were detected in 41 cases studied (26%) which is in line with the literature which states that the overall frequency of p53 mutations in breast cancer is approximately 20% [28]. Almost all the observed mutations locate in the highly conserved DNA-binding domain of the protein [18] (Table 2). Interestingly, our mutation analysis reveals that PILC is associated with a higher frequency (42.1%) of potentially pathogenic p53 mutations compared to ILC (13.6%). Even though some of these potentially pathogenic mutations (4 out of 11 mutations) do not result in an amino acid change, they have been reported before in different solid tumors including breast cancer [18]. These silent mutations are of particular interest. It has already been known for decades that non-transforming mutations can affect the protein production and therefore the function by interfering with various phases of transcription and translation [29]. Examples to possible scenarios are: i) interference with the editing of a gene transcript if silent mutations occur in codons that contain splicing enhancers responsible for the proper removal of introns, or ii) interference with the stability of mRNA by preventing correct folding; thus affecting the speed of translation of the protein as well as the degradation of the mRNA. Strikingly, Lamolle et al. recently showed that all reported non-transforming mutations of p53 which are documented in p53 somatic mutation database are preferentially located in conserved amino acid positions, which may depict their importance [30]. The majority of these mutations were found as single mutations, so never associated with other mutations in p53 gene, and they tended to be located inside suspected splicing enhancers. Interestingly, silent mutations observed in our study also exist as single mutations and locate either in an exposed residue of the DNA-binding domain or cause a predicted change in splicing. Moreover, silent mutations in p53 may also lead to a functional response by interfering with binding to MDM2. In this scenario, single silent point mutations in p53 mRNA can disrupt its binding to MDM2 resulting in aberrant p53 synthesis and degradation [31]. This failure in binding is shown to reduce p53 activity, thereby supporting the notion that silent p53 mutations can affect p53 function. Therefore, we think that our observation is noteworthy and in the light of recent discoveries in this field, assumptions on non-transforming mutations should be made carefully. We have also observed two previously reported and validated polymorphisms in a high number of our cases with no significant preference for either ILC variant. Of note is the fact that these polymorphisms were shown to be related with cancer susceptibility [18]. Especially the amino acid change 72P > R (Table 3) has been shown to be associated with breast cancer susceptibility [32–36].

The observation of this significantly increased frequency of pathogenic mutations in PILC -more than half of which do probably give rise to a non-functional protein based on predictions of computer models and for some based on literature (Table 2)- is also in line with mouse studies in which stochastic somatic inactivation of p53 and E-cadherin in the mammary gland induced the development of mouse PILC [16]. For some of the observed potentially pathogenic mutations, predictions on function of the protein were not available. However, it would not be unexpected if there were activating mutations among them since mutant p53 protein can also have a distinct function in cancer such as promoting invasion and metastases [37].

Another observation in this study was the significant correlation between p53 accumulation and transforming pathogenic mutations independent of any ILC variant. The positive correlation between p53 accumulation and mutations were made by other groups [4, 38–40] although not by all [41, 42]. It is apparent that the correlation between accumulation and mutations also depends on the influence of the mutation on the half-life of the protein. Most mutant forms of p53 have a longer cellular life and are therefore recognized by antibodies while others are not. This can explain our differential observation of p53 accumulation when all mutations are counted. A mutation might cause a misfolding of the protein structure (such as the above mentioned silent mutations resting in splicing enhancers or playing a role in mRNA stability) or a truncation that affects the epitope that is recognized by the antibody. We have also observed a case with p53 accumulation but no mutations. An explanation for this may be the possibility of mutations that are located outside of the investigated exons. Interestingly, a high percentage of p53-negative cases showed a polymorphism in residue 72, a mutation that has been implicated in enhanced targeting for degradation [43] providing a possible explanation for the p53 negativity in these cases. However, based on published data and our results, we can conclude that p53 immunostaining does not always reflect the genetic alteration and vice versa; the existence of a mutation does not always lead to p53 accumulation or complete lack of expression.

In conclusion, the clinically more aggressive pleomorphic variant of ILC bears a significantly higher frequency of p53 mutations compared to the classic variant, Moreover, since inactivation of p53 and E-cadherin in mice leads to the development of PILC, we envisage that p53 mutations may play a role in carcinogenesis of PILC.
